# Supplementary material for: The interactome of intact mitochondria by cross-linking mass spectrometry provides evidence for coexisting respiratory supercomplexes
Source: Mol Cell Proteomics. 2017 Dec 8;17(2):216–32. doi: 10.1074/mcp.RA117.000470 (PMC5795388; doi:10.1074/mcp.RA117.000470)
Supplement: Supplemental Data [file supp_RA117.000470_133922_0_supp_23978_fzffwf.zip › spectra_annotation/mito_DR_spectra_annotation/285-1-3-1-3-1.pdf]

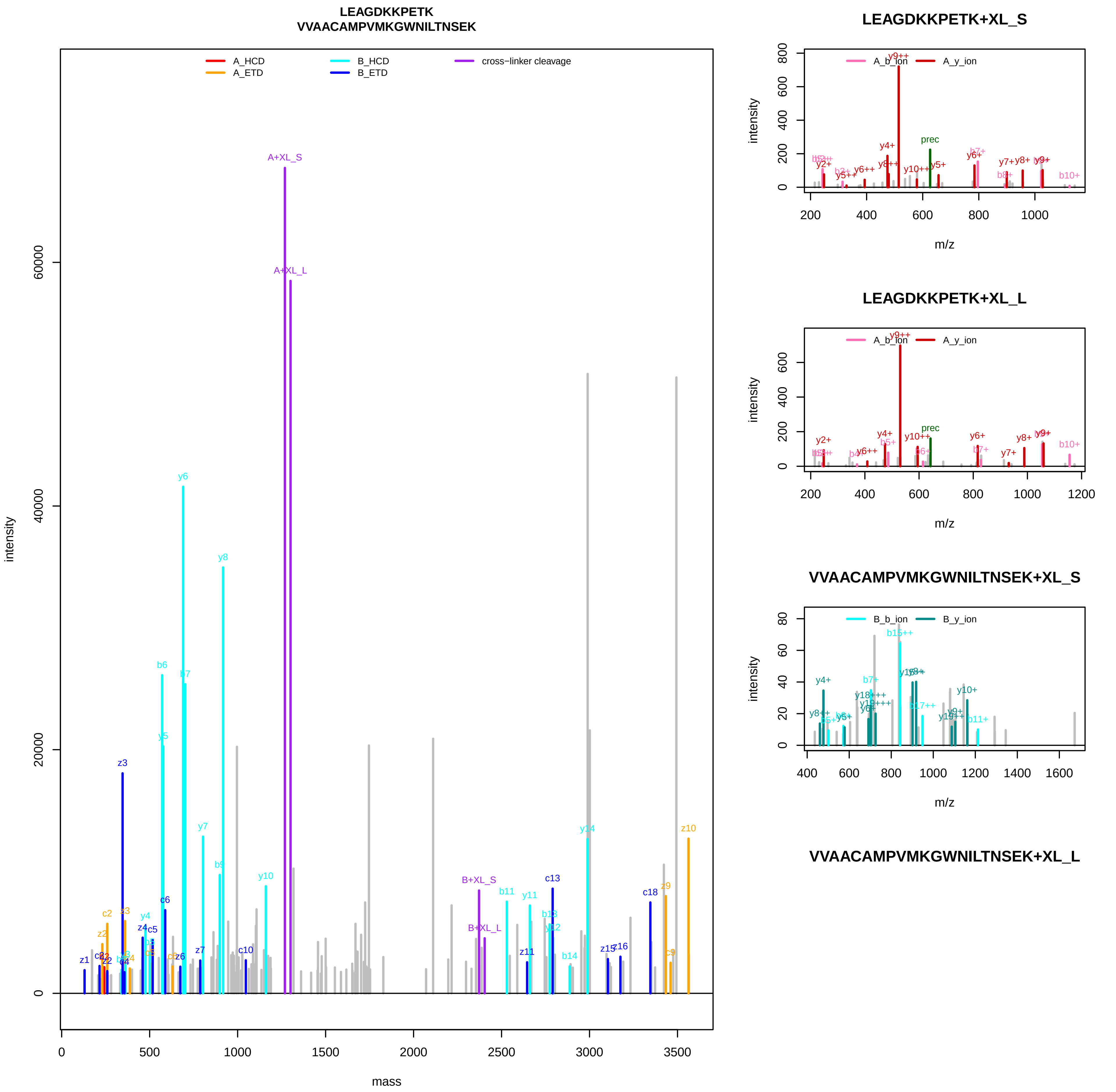
LEAGDKKPETK
VVAACAMPVMKGWNILTNSEK
A_HCD
A_ETD
B_HCD
B_ETD
cross−linker cleavage
A+XL_S
A+XL_L
B+XL_S
B+XL_L
y6
y8
b6
b7
y5
z3
y7
b9
y10
c6
c5
z4
y4
c2
z2
z3
z1
c4
z6
z7
c10
b11
y11
c13
z11
b13
y12
b14
y14
z15
z16
c18
z9
c9
z10
intensity
mass
0
20000
40000
60000
0
500
1000
1500
2000
2500
3000
3500
LEAGDKKPETK+XL_S
A_b_ion
A_y_ion
y9++
y4+
prec
y8++
y10++
y5+
y6+
b7+
y7+
y8+
y9+
b10+
y2+
y5++
y6++
intensity
m/z
0
200
400
600
800
1000
LEAGDKKPETK+XL_L
A_b_ion
A_y_ion
y9++
y2+
y4+
b5+
y6++
y10++
prec
b6+
y6+
b7+
y7+
y8+
y9+
b10+
intensity
m/z
0
200
400
600
800
1000
1200
VVAACAMPVMKGWNILTNSEK+XL_S
B_b_ion
B_y_ion
y4+
b15++
b7+
y18+++
y19+++
y8+
b17++
y10+
y9+
y19++
b11+
b5+
intensity
m/z
0
20
40
60
80
400
600
800
1000
1200
1400
1600
VVAACAMPVMKGWNILTNSEK+XL_L
